# Porf-2 Inhibits Neural Stem Cell Proliferation Through Wnt/β-Catenin Pathway by Its GAP Domain

**DOI:** 10.3389/fncel.2016.00085

**Published:** 2016-03-31

**Authors:** Guo-Hui Huang, Xi-Tao Yang, Kui Chen, Jin Xing, Lin Guo, Liang Zhu, Hong-Jiang Li, Xin-Cai Li, Sheng-Yi Zhang, Dong-Fu Feng

**Affiliations:** ^1^Department of Neurosurgery, Shanghai Ninth People's Hospital, Shanghai Jiao Tong University School of MedicineShanghai, China; ^2^Neuroscience Division, Department of Anatomy, Histology, and Embryology, Shanghai Jiao Tong University School of MedicineShanghai, China; ^3^Institute of Traumatic Medicine, Shanghai Jiao Tong University School of MedicineShanghai, China

**Keywords:** neural stem cell, porf-2, cell proliferation, wnt/β-catenin signaling, GAP

## Abstract

Neural stem cell (NSC) proliferation and differentiation play a pivotal role in the development of brain, the plasticity of the brain network, and the repair for brain function in CNS diseases. The mechanisms regulating NSC behavior are not well elucidated. Previous studies showed porf-2 functions as a modulator in central nerve system development. We here show that porf-2, a conserved family of RhoGAPs, is highly and specifically expressed in NSCs. We also demonstrate that porf-2 inhibits the proliferation of NSCs *in vivo and in vitro*, but has no effect on NSC differentiation. We investigated which domain is required for the role of porf-2 on NSC proliferation. By using neurosphere formation and Edu assay we confirmed the GAP domain is necessary for its function. In addition, we surveyed a few classical pathways on NSC proliferation and found that porf-2 inhibits NSC proliferation by suppressing the β-catenin nuclear translocation. Taken together, we show for the first time that porf-2 inhibits NSC proliferation through Wnt/β-catenin pathway by its GAP domain.

## Introduction

In adult central nervous system (CNS), neural stem cell (NSC) proliferation and differentiation play a pivotal role in the development of brain, the plasticity of the brain network, and the repair for brain function in CNS diseases (Kempermann and Gage, 1999; Snyder et al., 2001; Koehl and Abrous, 2011; Mongiat and Schinder, 2011; Marin-Burgin and Schinder, 2012; Varela-Nallar and Inestrosa, 2013; Bond et al., 2015). The multipotent NSCs are found mostly in the dentate gyrus (DG) of the hippocampus and the subventricular zone (SVZ) of the lateral ventricles in adult (Alvarez-Buylla and Garcia-Verdugo, 2002; Zhao et al., 2008). The NSCs have the capacity for self-renewal and multipotential differentiation (Reynolds and Weiss, 1992). Once activated, NSCs develop into proliferating intermediate progenitor cells and the undifferentiated neuroblasts that will finally maturate into neurons, astrocytes or oligodendrocytes (Reynolds and Weiss, 1992; McKay, 1997; Gage, 2000; Zhao et al., 2006; Ming and Song, 2011). The new born cells are able to integrate into the existing neural circuitry and contribute to different brain functions (Deng et al., 2010). Moreover, NSC transplantation is also a promising therapeutic strategy in several CNS diseases, including brain and spinal cord injuries, stroke, epilepsy, and neurodegenerative disease (Rossi and Cattaneo, 2002; Muller et al., 2006; Nakamura and Okano, 2013; Burns and Thapar, 2014; Aertker et al., 2015; Rolfe and Sun, 2015; Tong et al., 2015). Thus elucidating the molecular mechanism of NSC behavior will help us understand brain development and provide a platform for future NSCs' clinical application (Shimazaki, 2003; Selden, 2008).

To coordinate the proper progression of NSC proliferation and differentiation with preserving the stem cell pool, there should be a balance between the self-renewal and maintenance of NSCs and the differentiation. NSC proliferation and differentiation are a dynamic process and regulated by a variety of intrinsic and extrinsic factors including growth factors, cell surface receptors, signal transduction molecules, and transcription factors (Zhao et al., 2008; Bond et al., 2015). Several signaling molecules regulate NSC proliferation and differentiation including PTEN, Wnt, Notch, bone morphogenetic proteins (BMP), growth and neurotrophic factors, neurotransmitters, and transcription control (Suh et al., 2009; Ming and Song, 2011; Hsieh, 2012; Schwarz et al., 2012; Faigle and Song, 2013). However, our understanding of NSC behavior is still limited.

Preoptic regulatory factor-2 (Porf-2), also named as Cross GTPase-activating protein (CrossGAP)/Vilse, was first discovered from the preoptic area of the hypothalamus of the castrated male rat (Nowak, 1990, 2003). Nowark et al. found that porf-2 is widely expressed in diverse tissues including the CNS and peripheral tissues (Hu and Nowak, 1994; Nowak, 1997, 2003). In the CNS of the rat, it is highly expressed in the hippocampus, hypothalamus, and cerebral cortex (Hu and Nowak, 1994; Nowak, 2003). In drosophila, Hu et al. (2005) reported that porf-2 can directly interact with Roundabout (Robo) and regulate midline crossing of axon through Rac1-dependent cytoskeletal changes, which plays a significant role in the axon development of CNS. Similarly, Lundstrom et al. (2004) also reported that porf-2 is required for midline repulsion in CNS of the drosophila. In a recent study of neuronal development in mice, Lim et al. (2014) reported that porf-2 is involved in dendritic spine formation through Rac1 pathway. These data indicated diverse function of porf-2 in CNS. Additionally, in a study of porf-2 in NSCs, Ma et al. (Ma and Nowak, 2011) found that porf-2 may play a role in the proliferation of C17.2 cells, a mouse cerebellar multipotent cell line. But the detail function and mechanism of porf-2 on NSC proliferation and differentiation has not been elucidated yet.

Porf-2 has three domains: WW domain, Myosin Tail Homology 4 (Myth4) domain, and GTPase activating proteins (GAP) domain. The WW domain can bind to the Robo as reported (Lundstrom et al., 2004; Hu et al., 2005). The function of Myth4, which may provide a link between an actin-based motor protein and the microtubules cytoskeleton, is not yet fully understood (Kerber and Cheney, 2011). The GAP domain is predicted to transform Rac1/cdc42-GTP status to GDP status which is inactivated (Tcherkezian and Lamarche-Vane, 2007). But it still lacks of adequate evidence. Taken together, up to now, the function of the three domains is still rarely reported.

In the present study, we report that porf-2 is highly expressed in NSCs. We also demonstrate that porf-2 inhibits the proliferation of NSCs *in vivo and in vitro*. In addition it is the GAP domain that contributes to this phenomenon through the classical wnt/β-catenin pathway. Taken together, we show for the first time that porf-2, a conserved family of RhoGAPs, inhibits NSC proliferation through wnt/β-catenin pathway by its GAP domain.

## Materials and methods

### Isolation and culture of hippocampus NSCs

All procedures involving the use of laboratory animals were approved and monitored by the Animal Care Committee of the Shanghai Jiao Tong University School of Medicine. NSCs were isolated from newborn mice at postnatal day 0 or 1 as described previously (Yang et al., 2014). In brief, the newborn mice were terminated by decapitation and hippocampus were carefully dissected, pooled together in a 5 ml conical tube containing 1 ml dissection buffer (2 M NaCl, 1 M KCl, 1 M MgCl_2_, 155 mM NaHCO_3_,1 M Glucose, 108 mM CaCl_2_, 100 U/ml Penicillin/100 μg/ml streptomycin) and sliced into small pieces. After 5 min of settling, the supernatant was gently removed and the remaining tissue was incubated with 1,ml of 0.25% trypsin/0.03% EDTA at 37°C for 30 min followed by one time washing with Dulbecco's modified Eagle medium-DMEM/F12 (1:1) medium. After gentle trituration with a 1 ml pipette, the cell suspension was settled for 5,min, and carefully transferred to a new 15 mL tube. After centrifugation at 500 g for 5 min at room temperature (RT) and removal of the supernatant, the cell pellets were resuspended in 1 ml of complete NSCs medium, DMEM/F12 (1:1) medium supplemented with 1 × B27 (Gibco, 17504), 1 × N2(Gibco, 17502-048), plus 100 U/ml penicillin, 100 μg/ml streptomycin (Gibco, 15140-122), and basic fibroblast growth factor (bFGF) (20 ng/ml, R&D), epidermal growth factor (EGF; 20 ng/ml, R&D). NSCs were cultured for 4–5 days in the complete NSCs medium and formed neurospheres. When they reached 100–150 μm in diameter, the NSCs spheres were dissociated using 0.25% trypsin at 37°C and re-passaged. The experiments were performed using passage 3–6 neurospheres. Depending on the type of experiment, 1 to 2 × 10^5^ single cells in 2 ml of complete NSCs medium were plated into each well of a 6-well plate or 0.5–1 × 10^3^ cells in 500 ul of complete NSCs medium were plated into each well of a 24-well plate.

### Differentiation and proliferation of NSCs

For the differentiation experiment, the dissociated single NSCs suspension was plated in 24-well cell culture chambers, which had been coated by poly-D-lysine (Sigma-Aldrich, P7886) and 6 μg/mL laminin (Sigma-Aldrich, L4544). Then the cells were cultured for 7 days in the differentiating medium composed of NeurobasalA (Gibco, 10888) supplemented with 1 × B27 and 1% FBS. The differentiated cells were fixed for 30 min at room temperature in 4% paraformaldehyde (PFA) prior to immunostaining using antibodies specific for β-tubulin III (Tuj1; 1:1000, beyotime, AT809), glial fibrillary acidic protein (GFAP; 1:3000, Millipore, AB5806), and staining of nuclei with 4′,6-diamidino-2-phenylindole (DAPI). Each experiment average percentage of positive cells was calculated on randomly selected six fields of photograph.

To determine the proliferation of NSCs, Edu labeling was performed using a Click-iT® EdU Alexa Fluor® 647 Imaging Kit (Molecular Probes, life technology) according to the manufacturer's instructions. Briefly, primary neurospheres were dissociated into single cells and the dissociated single NSCs cell suspension was plated onto laminin-coated chamber slides. After 4 days culture in NSC complete medium, Edu labeling solution was added into the medium at final concentration of 10 μM. After 30 min incubation, attached NSCs were fixed with 4%PFA for 30 min, permeabilized, and then incubated with Click-iT® reaction cocktail 30 min at room temperature, protected from light. Chamber slides were then mounted with mounting medium with DAPI, and imaged with a confocal laser scanning microscope. Average percentage of positive cells was calculated on randomly selected six fields of photograph from slice. The Edu positive cells were counted by manually.

### Immunofluorescence

NSC spheres or the differentiated single neuronal cells on chamber slides were processed for histology as above. For brain slice immunofluorescence, after fixation with 4%PFA perfusion of mice, the brain slices were made for 30 μm per slice. Both the cells and the brain slice were blocked in 1 × PBS plus 0.3% Triton X-100 and 10% Normal Donkey Serum (Jackson ImmunoResearch, 122346) for 60 min at RT, and followed by incubation at 4°C for overnight in primary antibodies (diluted in blocking buffer). After 3 times washes (10 min for each wash) in 1 × PBS plus 0.1% Triton X-100, the sections or cells were incubated in the fluorescence-conjugated secondary antibodies (1:500 dilution in 1 × PBS) plus DAPI for 1 hr at RT. After 3 times washes, the chamber slides were then mounted with mounting medium and imaged.

Following primary antibodies were used: rabbit anti-Nestin (marker for NSPCs 1:400, Abcam), goat anti-Doublecortin (Dcx, marker for neuronal precursor cells, 1:100, Santa Cruz, sc8066), rabbit anti-Ki67 (1:200, Thermo scientific, RB-1510), rabbit anti-β-catenin (1:500, Cell Signaling Technology, 8480). Cell nuclei were stained with DAPI. Images were taken using a fluorescence microscope and analyzed with Image J.

Appropriate secondary antibodies were used as follows: donkey anti-mouse IgG conjugated with AlexaFluor 488, donkey anti-rabbit IgG conjugated with AlexaFluor 594, donkey anti-goat IgG conjugated with AlexaFluor 488, donkey anti-rat IgG conjugated with AlexaFluor 594, donkey anti-rabbit IgG conjugated with AlexaFluor 488, donkey anti-rabbit IgG conjugated with AlexaFluor 594. All of secondary antibodies were purchased from Molecular Probes (Life Technologies), and diluted 1:500 before use.

### Domain deleted DNA constructs

Porf-2 gene was amplified from hippocampal cDNA by PCR. Then the porf-2 gene was digested by Xbal1/Not1 enzyme and constructed to Plv-IRES-ZsGREEN1 vector. As for the domain deleted plasmid, the Δww-porf-2 (65-99 amino acid deleted), ΔMyth4-porf-2(717-902 amino acid deleted), ΔGAP-porf-2 (914-1099 amino acid deleted) were also constructed by PCR and ligased to Plv-IRES-ZsGreen vector. All the plasmids were packaged into lentivirus. The viruses were generated by co-transfection of two helper plasmids (pVSVG and pCMVΔ89) into the packaging cell line HEK293T at a ratio of 5:6:6 pLvx-porf-2:pVSVG:pCMVΔ89. Viruses were harvested 48 h after transfection by collecting the medium from transfected cells and stored at −80°C.

### Western blotting

Hippocampal regions from mice at different ages were dissected, homogenized, and solubilized at 4°C in Cell lysis buffer (P0013, beyotime) supplemented with 1 mM PMSF, 50 mM NaF, 1 mM Na3VO4 and protease inhibitor. NSCs were collected, homogenized, and solubilized in the same buffer. The total protein lysates were separated by SDS-PAGE and analyzed by western blotting with anti-GAPDH (1:5000, Sigma-Aldrich, G8795), mouse anti-Nestin (1:1000, Cell Signaling Technology, 4760), goat anti-Doublecortin (1:1000, Santa Cruz, sc8066), anti-p-PTEN(1:1000, Cell Signaling Technology, 9554s), anti-PTEN (1:1000, Cell Signaling Technology, 9188s), anti-β-actin (1:5000, Thermofisher scientific, MA5-15739) anti-p-Akt (1:1000, Cell Signaling Technology, 4060s), anti-Akt (1:1000, Cell Signaling Technology,2920s), p-mTOR (1:1000, Cell Signaling Technology, 5536s), anti-mTOR((1:1000, Cell Signaling Technology, 2972s), PI3K((1:1000, Cell Signaling Technology, 5569s), p-PI3k(1:1000, Cell Signaling Technology, 4228s), β-catenin (1:1000, Cell Signaling Technology, 8480), anti-NICD (1:1000, Cell Signaling Technology, 4147p), anti-HA (1:1000, Cell Signaling Technology, 2367s), anti-Histon H3 (1:3000, Cell Signaling Technology, 4499), anti-tuj1 (1:1000, beyotime, AT809), anti-porf-2 (1:1000, Santa Cruz Biotechnology, 87186). HRP-conjugated anti-rabbit, anti-mouse and anti-goat secondary antibodies (A0208, A0216 and A0181) were from beyotime. Analysis of the data was performed using NIH ImageJ software. The mean density of each band was normalized to actin or GAPDH signal in the same sample.

For the nuclear and cytoplasmic protein separated experiment, the protein in nucleus and cytoplasm were extracted following the Nuclear and Cytoplasmic Protein Extraction Kit (P0028, beyotime), then the same as above, the nuclear and cytoplasmic protein were analyzed by western blotting with indicated antibody.

### ShRNA viral production and infection

The sequence of porf-2 shRNA are as follows: shRNA1: CCCTTGATTCCTCATGAAT, ShRNA2: CTGCGAGATCTTCAAGCTA shRNA3: CAAAGTGACACAGCA CATA. Negative Control: TTCTCCGAACGTGTCACGT. All the shRNAs were constructed into PLKO.1 vector and the lentivirus were packaged as mentioned above.

The shRNA1 was also constructed to pAKD-CMV-bGlobin-mCherry-H1-shRNA vector. Packages of AAV9-shRNA viruses were provided by Obio Technology, Shanghai, China. Viral titers were 2.4 × 10^12^ particles/mL for AAV-ShRNA1, 1.3 × 10^13^ particles/mL for AAV-ShRNA-control.

For intracranial viral infection, 1 μl of concentrated AAV-shRNA was injected into the hippocampal DG area of P1 mice by stereotaxic injection. Six weeks after viral injection, the infected mice were sacrificed and processed for immunostaining as described above. The coordinates for the dentate gyrus were as follows: AP, 1 mm; ML, 1 mm; and DV, 1.5 mm.

### Statistical analysis

All experiments were performed at least three times in triplicate. The results are presented as mean ± SEM. Statistical differences were determined by Student's *t*-test for two-group comparisons or ANOVA followed by Tukey's test for multiple comparisons among more than two groups.

## Results

### Porf-2 is highly and specifically expressed in hippocampal NSCs *in vivo* and *in vitro*

**Figure 1:**
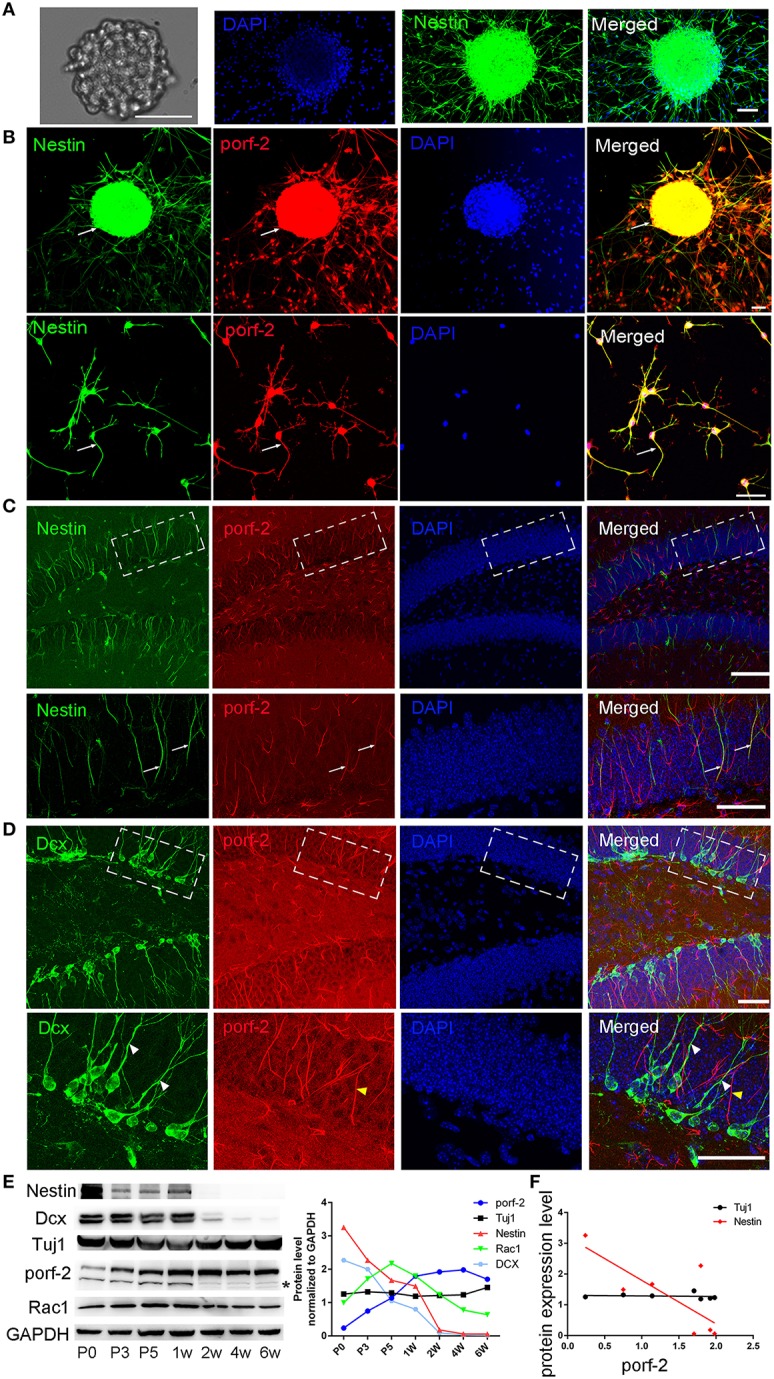
**Porf-2 is highly and specifically expressed in hippocampal NSCs *in vivo and in vitro*. (A)** Representative images of neurosphere formation after culture for 4–5 days. Identification of NSC neurosphere by immunofluorescence with nestin antibody. Scale bar: 50 μm. **(B)** Colocalization of porf-2 with nestin in neurosphere and single NSC visualized by immunofluorescence (indicated with white arrow). Scale bar: 50 μm. **(C)** Expression and colocalization of porf-2 with nestin visualized by immunolabeling with indicated antibody in DG area of hippocampus of brain slice. The boxes in upper panel are shown at higher magnification in lower panels. Whtie arrow indicates the colocalization of porf-2 with nestin. Scale bars: 100 μm in the upper panel and 50 μm in the lower panel. **(D)** No colocalization of Porf-2 with DCX in DG area. The boxes in upper panel are shown at higher magnification in lower panels. White arrowhead indicates DCX positive cell. Yellow arrowhead indicates porf-2 positive cell. Scale bars: 50 μm in both upper and lower panels. **(E)** The expression pattern of porf-2, nestin, DCX, Tuj1 and Rac1 proteins were analyzed by western blotting of hippocampal lysates at postnatal day 0, 3, 5; 1, 2, 4, and 6 week. The same blot was probed with anti-GAPDH antibody as a loading control. The symbol ^*^ indicates non-specific signal in western blot analysis. Quantification of porf-2, nestin, DCX, Tuj1, and Rac1 protein level at indicated time. **(F)** The expression level of porf-2 was correlated with nestin (red dots) but not tuj1 (black dots) during development. (Red dot, *r*^2^ = 0.5785, *p* < 0.05. Black dot, *r*^2^ = 0.078, *p* > 0.05).

To identify whether porf-2 is expressed in NSCs, we cultured primary hippocampal NSCs. After 5 days culture, NSCs grows into neurosphere stage (Figure 1A). Most of cells in such neuroblasts were immunostained positive for nestin, a common NSC maker (Figure 1A). Then we performed immunofluorescence on the cultured NSC neurosphere and single NSC with porf-2 and nestin antibody. Immunofluorescence assay with these antibodies revealed that endogenous porf-2 is localized in the nucleus and fiber of the NSCs, as indicated by its colocalization with nestin (Figure 1B). Consistent with *in vitro* study, porf-2 colocalized with nestin in the DG area of the hippocampus (Figure 1C). Moreover, porf-2 was also expressed in GFAP^+^/Sox-2^+^ cells, further confirming its expression in NSCs (Figure S1). While, interestingly, there is no localization of porf-2 with DCX, a progenitor cell maker (Figure 1D). This indicted that porf-2 is specifically expressed in the NSCs.

To examine the expressing pattern of porf-2 during development of NSCs, we performed western blot on hippocampus tissue lysate throughout early postnatal development. As shown in Figure 1E, porf-2 gradually increased during development and in a time-dependent manner. Also the protein level of nestin and DCX gradually decreased (Figure 1E), which is consistent with the fact that in the early stage there are more NSCs in DG area. Then we analyzed the correlation between porf-2 and nestin expression and the result showed that there was a negative correlation (*p* < 0.05, *r*^2^ = 0.5785; Figure 1F), indicating that porf-2 correlated with nestin expression during the development. Meanwhile porf-2 had no correlation with Tuj1 (*r*^2^ = 0.078, *p* > 0.05; Figure 1F). The contrary expression pattern indicted porf-2 may have a negative role on the nestin expression. Together these data demonstrate porf-2 is specifically expressed in the NSCs *in vitro and in vivo* and may have a negative correlation with nestin expression.

### Knockdown of porf-2 promotes NSC proliferation, but has no effect on NSC differentiation *in vitro*

**Figure 2:**
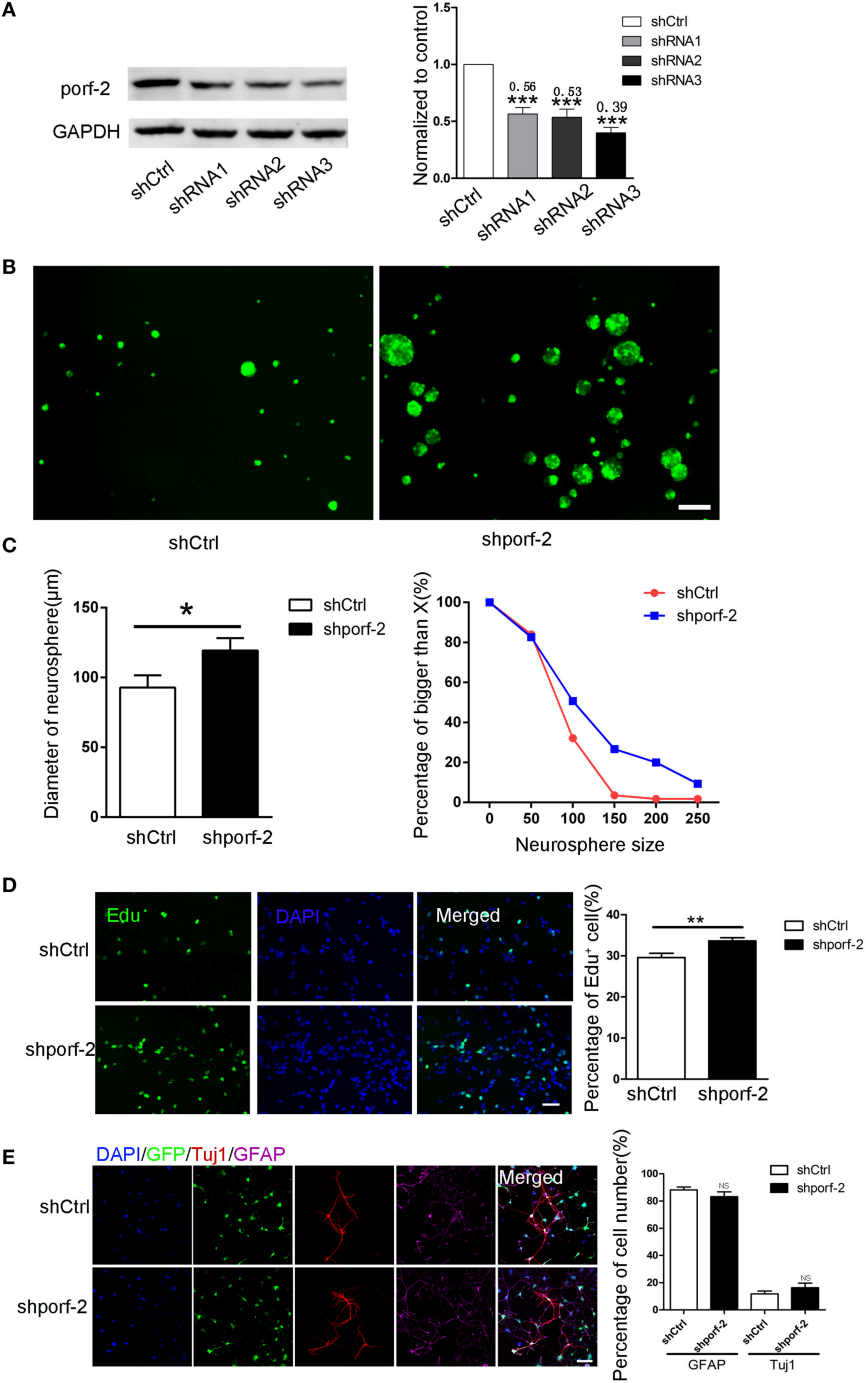
**Knockdown of porf-2 increased NSC proliferation but had no effect on NSC differentiation. (A)** Knockdown of porf-2 by lenti-shRNA infection was confirmed by immunoblot analysis in NSCs. ^***^P < 0.001 vs. shCtrl. **(B)** Representative image of neurosphere after 4 days culture in lenti-shCtrl and lenti-shporf-2 group. Scale bar: 200 μm. **(C)** Comparison and Quantification of the diameter of NSC spheres between shCtrl and shporf-2 group after 4 days lentivirus infection. The percentage of different neurosphere size was quantified (right panel). Data are mean ± SEM (*n* = 4). ^*^p < 0.05. **(D)** Representative image of Edu positive cells in shCtrl and shporf-2 group. Quantification of the percentage of Edu positive cell number in each group. The number of Edu positive cells is normalized to the total cell number. Scale bar: 100 μm. Data are mean ± SEM (*n* = 4). ^**^p < 0.01. **(E)** Confocal images showing the Tuj1 and GFAP positvie cell after 7 days differentiation of NSCs in each group. Quantification of the percentage of Tuj1 and GFAP positive cell number in shCtrl and shporf-2 group. The number of Tuj1 or GFAP positive cells is normalized to the total cell number. Scale bar: 100 μm. Data are mean ± SEM (*n* = 4). NS: no significant difference *P* > 0.05.

We have described porf-2 expression pattern. Next, we explored its function on NSC behavior. Firstly, we performed knockdown of porf-2 in mouse hippocampal NSCs by using lentiviral-mediated shRNA delivery. As shown in Figure 2A and Figure S2, all the shRNAs have a high knockdown efficiency, especially the shRNA3, about 61% knockdown in protein level (Figure 2A). Thus we use shRNA3 to investigate the effect of porf-2 on NSCs for all the subsequent testings.

To assess the role of porf-2 on NSC proliferation, we performed neurosphere formation assay as described in methods. Four days after the lenti-shRNA infection, all NSC neurospheres were GFP-postive in both groups (Figure 2B). By quantifying the size of multipotent neurosphere, we found that knockdown of porf-2 resulted in larger size neurosphere than those of controls (Figures 2B,C). Moreover, the percentage of large size neurosphere in shporf-2 group is higher in comparison to control (Figure 2C). These data indicated that knockdown of porf-2 can promote NSC proliferation. The effect of porf-2 on NSC proliferation was further measured by the Edu assay. Consistent with the results in neurosphere assay, knockdown of porf-2 showed higher number of Edu positive cells than the control cells, which means NSC proliferation was enhanced by porf-2 knockdown (Figure 2D). Together, these results indicate that knockdown of porf-2 has a promoting effect on NSC proliferation.

To study the possible effect of porf-2 on NSC differentiation, we induced the cell differentiation in NSC differentiation medium for 7 days after lenti-shRNA infection. The cells in single cell layer were then fixed prior to routine histological process. The NSC differentiation into mature neural cells was further verified by immunostaining, using antibodies of neural cell lineage markers, including Tuj1 (neurons), GFAP (astrocytes) and the cell nuclei were labeled with DAPI (Figure 2E). The percentage of Tuj1 and GFAP labeled cells was calculated and no significant difference was observed between the two groups (Figure 2E). Overall, our results suggest that knockdown of porf-2 can improve NSC proliferation but has no role on NSC differentiation.

### Porf-2 inhibits NSC proliferation *in vitro*

**Figure 3:**
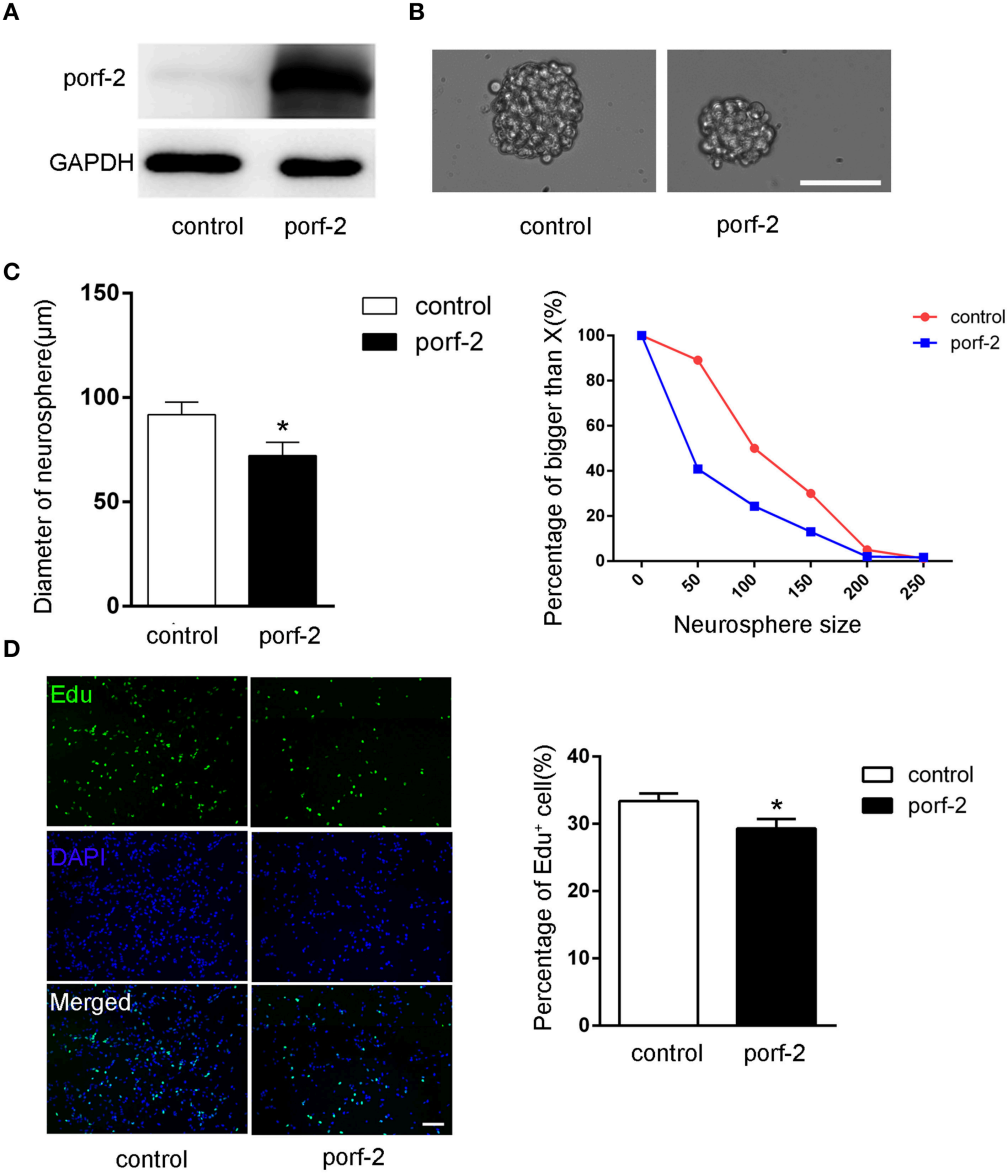
**Porf-2 inhibits NSC proliferation *in vitro*. (A)** Confirming the overexpression of porf-2 protein by immunoblot. **(B)** The neurosphere morphology in control and porf-2 overexpression group. Scale bar: 50 μm. **(C)** Quantification of the diameter of neurosphere in each group. Data are mean ± SEM (*n* = 3). **(D)** Representative image of Edu positive cells in each group. The total NSC number was counted by DAPI. Quantification of the percentage of Edu positive cell number in each group. Scale bar: 100 μm. Data are mean ± SEM (*n* = 4). ^*^P < 0.05.

To further demonstrate the role of porf-2 on NSC proliferation, we overexpressed porf-2 in NSCs. The overexpression of porf-2 was confirmed by WB (Figure 3A). By using the neurosphere assay, we measured the diameter of NSC sphere and found that overexpression of porf-2 can significantly reduce the diameter of NSC sphere compared to control (Figures 3B,C). In the Edu assay, as shown in Figure 3D, ~34% NSCs in control and about 28% NSCs in porf-2 overexpression group were Edu positive, confirming that overexpression of porf-2 decreased NSC proliferation. Altogether, our results suggest that overexpression of porf-2 inhibits NSC proliferation *in vitro*.

### Porf-2 inhibits NSCs proliferation through its GAP domain

**Figure 4:**
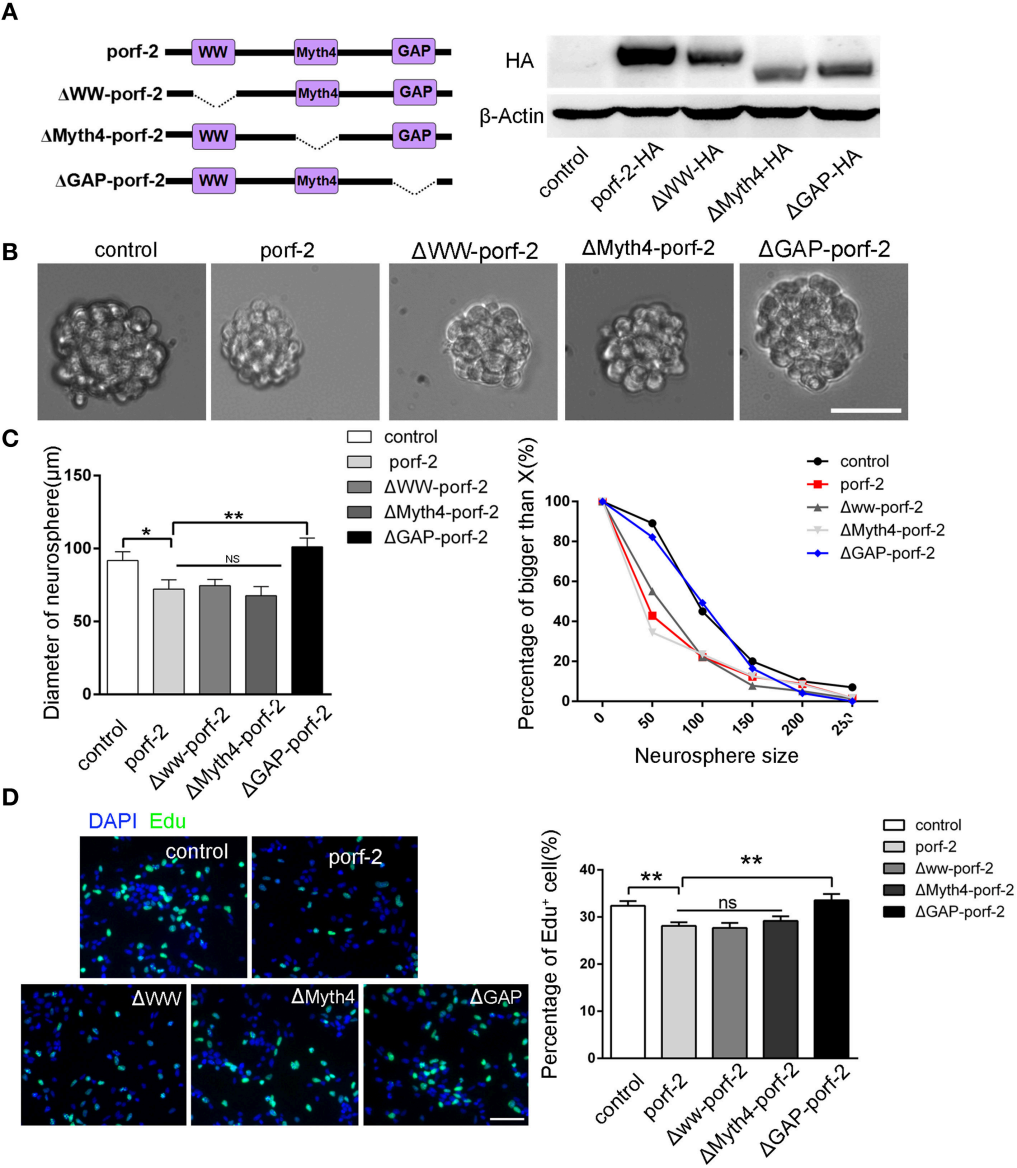
**The GAP domain is required for the porf-2's function on NSC proliferation. (A)** The construct pattern of the domain deleted plasmid. Verification of domain deleted plasmid by immunoblot. The same blot was probed with β-actin antibody as a loading control. **(B)** The morphology of neurosphere in different groups. Scale bar: 50 μm. **(C)** Quantification of neurosphere size in each group. ^*^p < 0.05, ^**^p < 0.01. **(D)** Representative images of Edu positive cells in indicated group. Quantification of the percentage of Edu positive cell number in each group. Scale bar: 50 μm. Data are mean ± SEM (*n* = 5). ^**^P < 0.01.

As porf-2 has three domains: WW, Myth4, and GAP domain, we tried to find out which domain is required in the NSC proliferation. Next, we created three domain deleted plasmids of porf-2. The construct pattern was shown in Figure 4A. Our western blot result suggested that the three plasmids were successfully constructed (Figure 4A). In agreement with our result above, overexpression of porf-2 showed smaller neurosphere phenotype compared to control (Figures 4B,C). Similarly, overexpression of Δww-porf-2 and ΔMyth4-porf-2 showed smaller neurosphere phenotype compared to control, which is consistant with porf-2 overexpression. While interestingly, the diameter of neurosphere in ΔGAP-porf-2 group was back to control level, which indicated that the GAP domain is the functional domain of porf-2 in NSC proliferation (Figures 4B,C).

In agreement with the above-mentioned neurosphere formation assay, overexpression of porf-2, Δww-porf-2, or ΔMyth4-porf-2 showed lower percentage of Edu positive cells in Edu assay, and ΔGAP-porf-2 showed no difference in comparison to control (Figures 4C,D). The result indicated that ΔGAP-porf-2 lost its function on NSC proliferation. In other words, the GAP domain is required for porf-2's function on NSC proliferation. Above all, porf-2 inhibits NSC proliferation and the GAP domain plays an essential role in this process.

### Porf-2 inhibits the NSC proliferation through Wnt/β-catenin pathway

**Figure 5:**
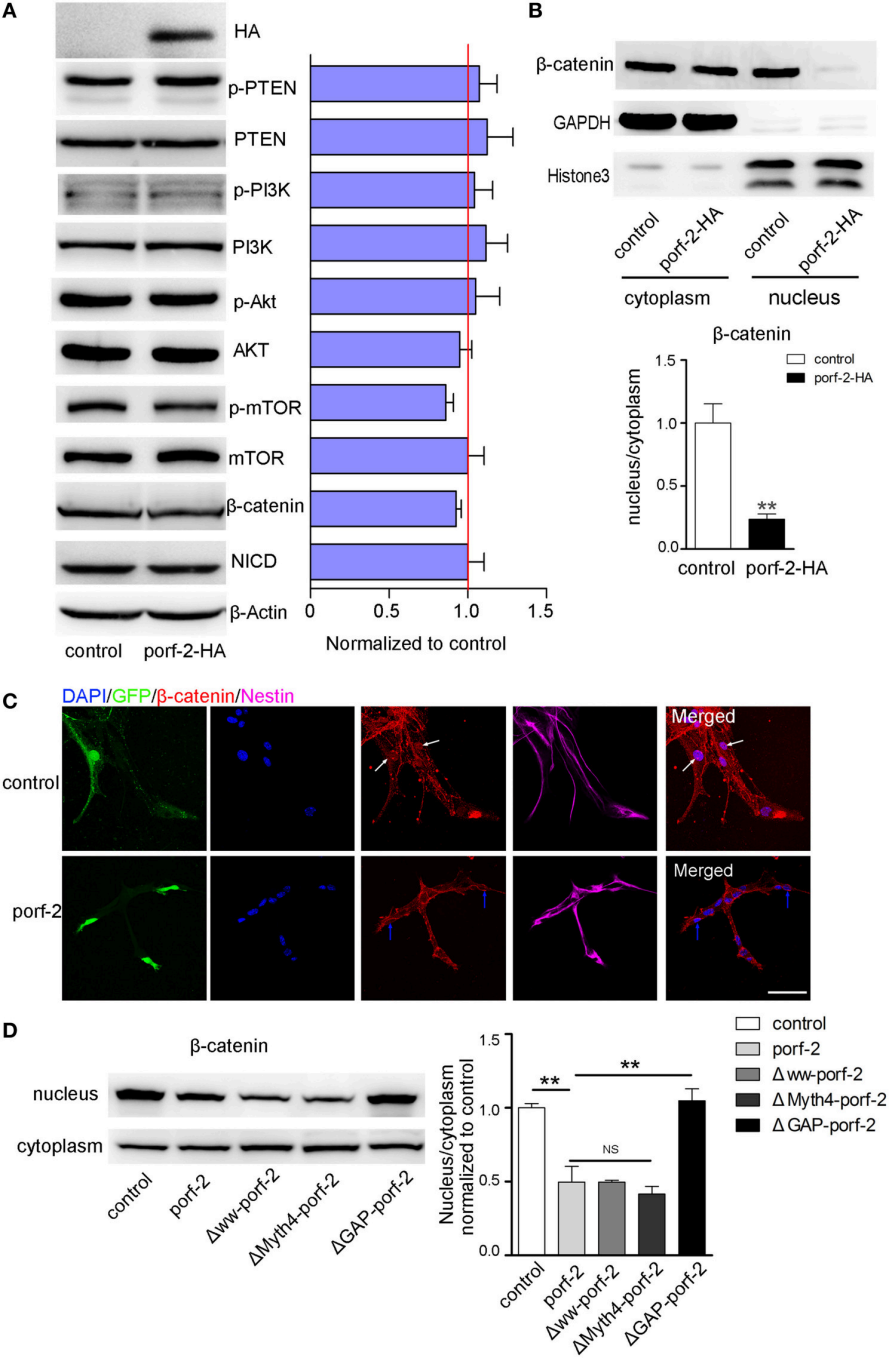
**Porf-2 inhibits NSC proliferation through wnt/β-catenin pathway. (A)** The expression and quantification of p-PTEN, PTEN, p-PI3K, PI3K, p-Akt, Akt, p-mTOR, mTOR, NICD, and β-catenin protein in control and porf-2 overexpression group. Data are mean ± SEM (*n* = 3). β-actin was used as a loading control. **(B)** The expression and quantification of β-catenin protein in cytoplasm and nucleus in control and porf-2 overexpression group. GAPDH and HistoneH3 were used as loading controls for protein in cytoplasm and nucleus respectively. ^**^p < 0.01. **(C)** Confocal microscopy image of β-catenin expression in NSCs after lentivirus infection. White arrow indicated the expression of β-catenin in nucleus in control group. Blue arrow indicated the expression of β-catenin in nucleus in porf-2 overexpression group. Scale bar: 50 μm. **(D)** The expression and quantification of β-catenin protein in cytoplasm and nucleus in different groups. Data are mean ± SEM (*n* = 3). ^**^p < 0.01.

**Figure 6:**
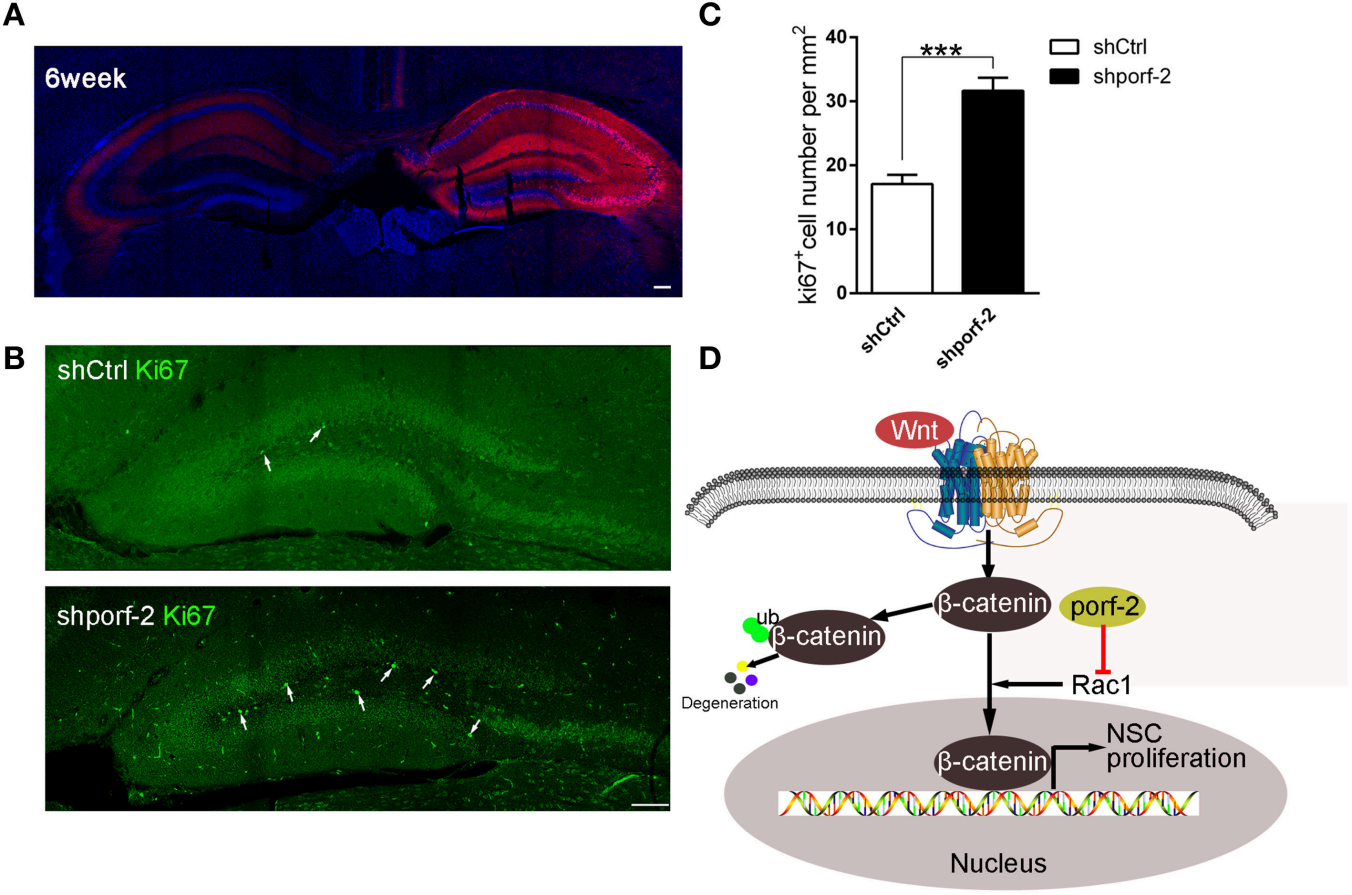
**Knockdown of porf-2 promotes NSC proliferation *in vivo*. (A)** The expression of AAV-shRNA indicated by mcherry after 6 weeks infection. Scale bar 100 μm. **(B)** The ki67 staining in brain slice in each group. The white arrow indicated ki67 positive cell. Scale bar: 100 μm. **(C)** Quantification of ki67 positive cell number in each group. Data are mean ± SEM (*n* = 8). ^***^P < 0.001. **(D)** Schematic drawing demonstrating a previously uncharacterized mechanism for porf-2 inhibits NSC proliferation through wnt/β-catenin pathway by its GAP domain.

As is well-known, there are a few classical pathways involved in NSC proliferation: PTEN/PI3k-Akt, Akt-mTOR, Notch, and Wnt/β-catenin pathway (Liu and Niswander, 2005; Egeland et al., 2015). To understand the mechanism of how porf-2 inhibited NSC proliferation, we surveyed above pathways. As shown in **Figure 6A**, p-PTEN/PTEN, p-PI3K/PI3K, p-Akt/Akt protein level remained unchanged after porf-2 overexpression, indicating that porf-2 had no effect on PTEN/PI3K/Akt pathway. Then we found that although p-mTOR protein level had a slight decrease, it showed no significant difference with control (Figure 5A). We also checked the Notch and Wnt/β-catenin pathway and found that there was no difference in NICD and β-catenin protein level between overexpression of porf-2 and control group (Figure 5A). Similar results were obtained when porf-2 was knock down in NSCs (data not shown).

Given the fact that β-catenin plays a pivotal role in Wnt/β-catenin pathway by entering into nucleus to mediate NSCs proliferation, a process known as β-catenin nuclear translocation (Kahn, 2014; Sun and Hevner, 2014), we analyzed the effect of porf-2 on the cellular distribution of β-catenin. The cytoplasm and nucleus proteins were separated as indicated by GAPDH and HistoneH3 (Figure 5B). Interestingly, we found that though there was no change of β-catenin in the cytoplasm, the protein level of β-catenin in nucleus significantly decreased after porf-2 overexpression, which suggested porf-2 decreased the translocation of β-catenin to nucleus (Figure 5B). Consistent with the western blot result, we observed that β-catenin mostly expressed in cytoplasm instead of nucleus after porf-2 overexpression by immunoflourence (Figure 5C). Also we investigated the effect of mutant porf-2 plasmids on β-catenin nuclear translocation. As shown in Figure 5D, the protein level of β-catenin in nucleus significantly decreased in Δww-porf-2 and ΔMyth4-porf-2, which is consistant with porf-2 overexpression, while the protein level of β-catenin in nucleus in ΔGAP-porf-2 group was back to normal. This suggested that ΔGAP-porf-2 domain is responsible for porf-2′s effect on β-catenin nuclear translocation (Figure 5D). Based on these findings, we propose that porf-2 can decrease the nuclear translocation of β-catenin through its GAP domain.

### Knockdown of porf-2 promotes NSC proliferation *in vivo*

To further investigate the function of porf-2 on NSC proliferation *in vivo*, we took advantage of AAV-mediated shRNA delivery. At postnatal day 1, the AAV-shRNA was injected into the DG area of unilateral hippocampus. Six weeks later, the whole ipsilateral hippocampus area was infected with the AAV virus, indicated by red fluorescence (Figure 6A). To assess the effect of shporf-2 on NSC proliferation, the infected brain slices were stained with an antibody against the proliferation maker ki67. Next we calculated the ki67 positive cell number in the DG area and found that the number of ki67 positive cells was higher in shporf-2 group in comparison to control (Figures 6B, C). Our results suggest that knockdown of porf-2 can promote NSC proliferation *in vivo*.

## Discussion

Our study is the first to show that porf-2 is highly and specifically expressed in hippocampal NSCs. It also palys a pivotal role in NSC proliferation *in vivo and in vitro*. Moreover, our findings clearly indicate that porf-2 inhibits NSCs proliferation through its GAP domain by Wnt/β-catenin pathway (Figure 6D).

Previous studies showed porf-2 functions as a key modulator in axon and dendrite spine development. In CNS, porf-2 is expressed in different regions of brain, hippocampus, cerebral cortex, hypothalamus, and so on (Hu and Nowak, 1994; Nowak, 1997, 2003). However, whether or not it is expressed in hippocampal NSCs and its expression pattern are not elucidated. Here in this study, we took advantage of immunoflourenscence and western blot to first demonstrate porf-2 is highly expressed in NSCs and its expression increases in a time-dependent manner. These findings prompt us to further investigate its function on NSC behavior.

NSCs are a unique population of cells that exhibit stem cell properties, including self-renewal, i.e., production of a large number of progeny, and multipotency, i.e., differentiation into the three CNS neural lineages (Louis and Reynolds, 2005). Because NSCs have the ability to support neurogenesis throughout adulthood, they have been viewed as a renewable source of neural precursors for regenerative transplantation in various CNS diseases (Horner and Gage, 2000; Teng et al., 2002). Increasing evidence suggests that NSCs go through a process of self-renewal, proliferating, and differentiating into the appropriate lineage when inflammatory damage or injury occurs in the nervous system (Pluchino and Martino, 2008). In the present study we demonstrate porf-2 can inhibit NSC proliferation and knockdown of porf-2 promotes NSC proliferation, which provides us a new insight into neurogenesis. Unexpectedly, our study showed that porf-2 did not affect NSC differentiation, that is to say, it isn't involved in NSC differentiation into neuron or astrocyte. The possible reason is that porf-2 is specifically expressed in activating NSCs but not the later stages indicated by DCX, which means once the NSCs go into differentiation stage, porf-2 is not expressed any more. Thus, porf-2 cannot exert its functions.

Ma et al. (Ma and Nowak, 2011) has recently reported that knockdown of porf-2 induced increased proliferation and drug-induced apoptosis through p53/p21 pathway in C17.2 cell. But Ma's report on NSC proliferation is based on an artificial stem cell line (Ma and Nowak, 2011). In addition, the porf-2 protein reported in Ma's study is a 65KD peptide (Ma and Nowak, 2011), not the full-length porf-2, which has a few transcripts. Different with Ma's study, we focused on the full length of porf-2 gene, which encodes a 130 kd protein. Instead of studying stem cell line, we paid attention to cultured hippocampal NSCs and observed knockdown of porf-2 significantly increased the size of neurosphere and the number of Edu positive cells. The results indicated that downexpression of porf-2 promoted NSC proliferation. Consistent with the down-expression phenotype, overexpression of porf-2 decreased the size of neurosphere and the percentage of Edu positive cell number. Moreover, AAV-shRNA-porf-2 injection increased the ki67 positive cell number in DG area, which further confirmed the function of porf-2 *in vivo* assay.

It is known that porf-2 has three conserved domains. The WW domain of porf-2 was reported as a binding domain which can bind to member receptor robo and scaffold protein CNK2 to transduce signal pathway to mediate axon and spine development (Hu et al., 2005; Lim et al., 2014). The MyTH4 domain has been identified as a conserved domain in the tail domains of several different unconventional myosins for intracellular trafficking, cell division, and muscle contraction (Lu et al., 2014). The GAPs, which enhance the intrinsic GTPase activity, leading to the inactive state of the GTPase, involved in multiple cellular process including NSC proliferation (Dasgupta and Gutmann, 2005; Tcherkezian and Lamarche-Vane, 2007). But as for porf-2, which domain is necessary for its function on NSCs is not clear. In our *in vitro* neurosphere formation and Edu assay, we found that the full-length-porf-2, Δww-porf-2, the ΔMyth4-porf-2 all showed a smaller size of neurosphere and lower percentage of Edu positive cell number, while the ΔGAP-porf-2 showed no difference compared to control. Furthermore, compared to full-length-porf-2 group, the ΔGAP-porf-2 showed a larger size of neurosphere and higher percentage of Edu positive cell number. The data showed that the GAP domain deleted porf-2 lost its inhibitory function on NSC proliferation.

The basic action of NSC proliferation is mediated by several signal pathways: PTEN/Akt, Akt/mTOR, Notch, and Wnt/β-catenin pathway (Suh et al., 2009). In our study, we found that porf-2 overexpression didn't affect the protein expression of p-PTEN, p-Akt, p-mToR, which implied that porf-2 may not be involved in PTEN/Akt/mTOR signaling pathway. The Notch signaling pathway is a highly conserved cell signaling system involved in NSC proliferation and differentiation (Yoon and Gaiano, 2005; Louvi and Artavanis-Tsakonas, 2006; Ables et al., 2011; Homem et al., 2015). Ligand proteins binding to the extracellular domain of Notch induced proteolytic cleavage and release of the Notch intracellular domain (NICD), which translocated to the nucleus to activate transcription of downstream target genes to mediate NSC proliferation and differentiation (Louvi and Artavanis-Tsakonas, 2006). We examined the NICD protein level and found that porf-2 overexpression didn't affect the NICD expression, which suggested that porf-2 didn't affect the Notch signaling pathway.

Although these above-mentioned signaling pathways aren't involved in porf-2′s action on NSC proliferation, we demonstrated porf-2 inhibits NSC proliferation through the β-catenin pathway. In present study, we found that overexpression of porf-2 didn't affect the total β-catenin protein level, but reduced the nuclear β-catenin level. By using immunofluorescence, we also confirmed that porf-2 overexpression suppressed β-catenin nuclear translocation. Porf-2 has been reported to mediate midline axon guidance by inactivating Rac1 in the developing stage (Lundstrom et al., 2004; Hu et al., 2005). It was reported that Rac1 can mediate β-catenin nuclear translocation (Esufali and Bapat, 2004; Upadhyay et al., 2008; Wu et al., 2008; Bosco et al., 2010). Moreover porf-2 is a RhoGAP, which can inactivate Rac1 as reported (Lundstrom et al., 2004; Hu et al., 2005; Lim et al., 2014). Thus we propose that porf-2 can mediate β-catenin nuclear translocation through inactivating Rac1 by its GAP domain. To test this hypothesis, we deleted the GAP domain and found that the ΔGAP-porf-2 lost its function on β-catenin nuclear translocation. To further confirm our hypothesis, we performed the Edu assay to verify the effect of Rac1 DN on NSC proliferation. And both the Rac1 DN and porf-2 overexpression showed a lower percentage of Edu positive cell number in comparison to control (Figure S3), suggesting Rac1 DN and porf-2 overexpression showed the same phenotype in NSC. Thus, our work revealed an uncovered mechanism through which porf-2-induced proliferation impairment takes place by the modulation of Wnt/β-catenin signaling through its GAP domain, which may inactivate Rac1 and result in the suppression of β-catenin nuclear translocation. However, the precise mechanism underlying porf-2-induced alteration of Wnt/β-catenin signaling needs to be fully elucidated.

As is reported in previous studies, GABAAR-mediated transmission regulates multiple steps of adult neurogenesis, including control of stem/precursor cell proliferation, cell fate decision, migration of precursor cells, survival of immature neurons, dendritic growth, and synaptogenesis (Platel et al., 2007; Ming and Song, 2011; Dieni et al., 2012). In the SGZ, NSCs respond tonically to GABA via the α5β3γ2 GABAAR composition to control their quiescent condition (Song et al., 2012). It is also reported that GABAARs containing the α4 subunit are expressed in typeI NSCs to control their proliferation rate (Duveau et al., 2011). Besides, Liu et al. reported that both the inter-neuron in the stem cell niche and the neuroblasts itself can release GABA, leading to tonic GABAAR activation of neural precursors and a decrease in proliferation (Liu et al., 2005). Still it is not clear how the signaling through GABAAR is processed in mediating NSC behavior. Interestingly, in our current study, we uncovered that a novel RhoGAP protein, porf-2 plays a inhibitory effect on NSC proliferation, suggesting its intrinsic role in maintaining stem cell quiescence as well as GABAAR. However, there are still questions unsolved. What's the upstream proteins or signals of porf-2? How is porf-2 regulated in the brain development or neurogenesis? Is it possible that GABAAR may function via Porf-2 and Rac1? Further study are needed to investigate whether porf-2 may serve as a downstream effector of GABAAR or other membrane receptors to inhibit NSC proliferation.

In summary, our study demonstrates for the first time that porf-2 can inhibit NSC proliferation but has no effect on neuron/astrocyte differentiation of NSCs. And porf-2 inhibits NSC growth through its GAP domain by Wnt/β-catenin pathway. From this point of views, the porf-2 mediated signal pathway may provide us a new insight into its future applications in nerve system.

## Author contributions

GH performed the experiments of morphology, histology, and biochemistry. GH, XY, KC, JX, and LG has been involved in analysis and interpretation of data and in drafting and revising the manuscript critically. LZ, HL, XL, and SZ set up the platform for AAV virus experiments and supervised the morphological analysis. DF designed experiments and revised the manuscript critically. All authors agree that all the questions related to the accuracy or integrity of the paper have been appropriately investigated and resolved, giving final approval of the version to be published.

### Conflict of interest statement

The authors declare that the research was conducted in the absence of any commercial or financial relationships that could be construed as a potential conflict of interest.

## Supplementary material

Figure S1 **Porf-2 is expressed in NSCs of subgranular zone in DG**. Expression of of porf-2, Sox-2, GFAP were visualized by immunolabeling with indicated antibody in DG area of brain slice. The boxes in left panel are shown at higher magnification in right panels. The white arrowhead indicated the colocalization of porf-2 with GFAP^+^/Sox-2^+^ cell. Scale bars: 25 μm in the left panel and 10 μm in the right panel.

Figure S2 **Porf-2 was knocked down by shRNA. Porf-2 knock-down was confirmed by Western Blot analysis**. The symbol^*^ indicated the non-specific band smaller than 120 KD. GAPDH was used as a loading control.

Figure S3 **Rac1 DN showed the same phenotype as Porf-2 expression on NSC proliferation**. Representative image of Edu positive cells in each group. The total NSC number was counted by DAPI. Quantification of the percentage of Edu positive cell number in each group. Scale bar: 50 μm. Data are mean ± SEM (*n* = 4). ^**^*P* < 0.01 vs. control.
