# Supplementary figures and images for: Porf-2 Inhibits Neural Stem Cell Proliferation Through Wnt/β-Catenin Pathway by Its GAP Domain
Source: Front Cell Neurosci. 2016 Mar 31;10:85. doi: 10.3389/fncel.2016.00085 (PMC4814557; doi:10.3389/fncel.2016.00085)

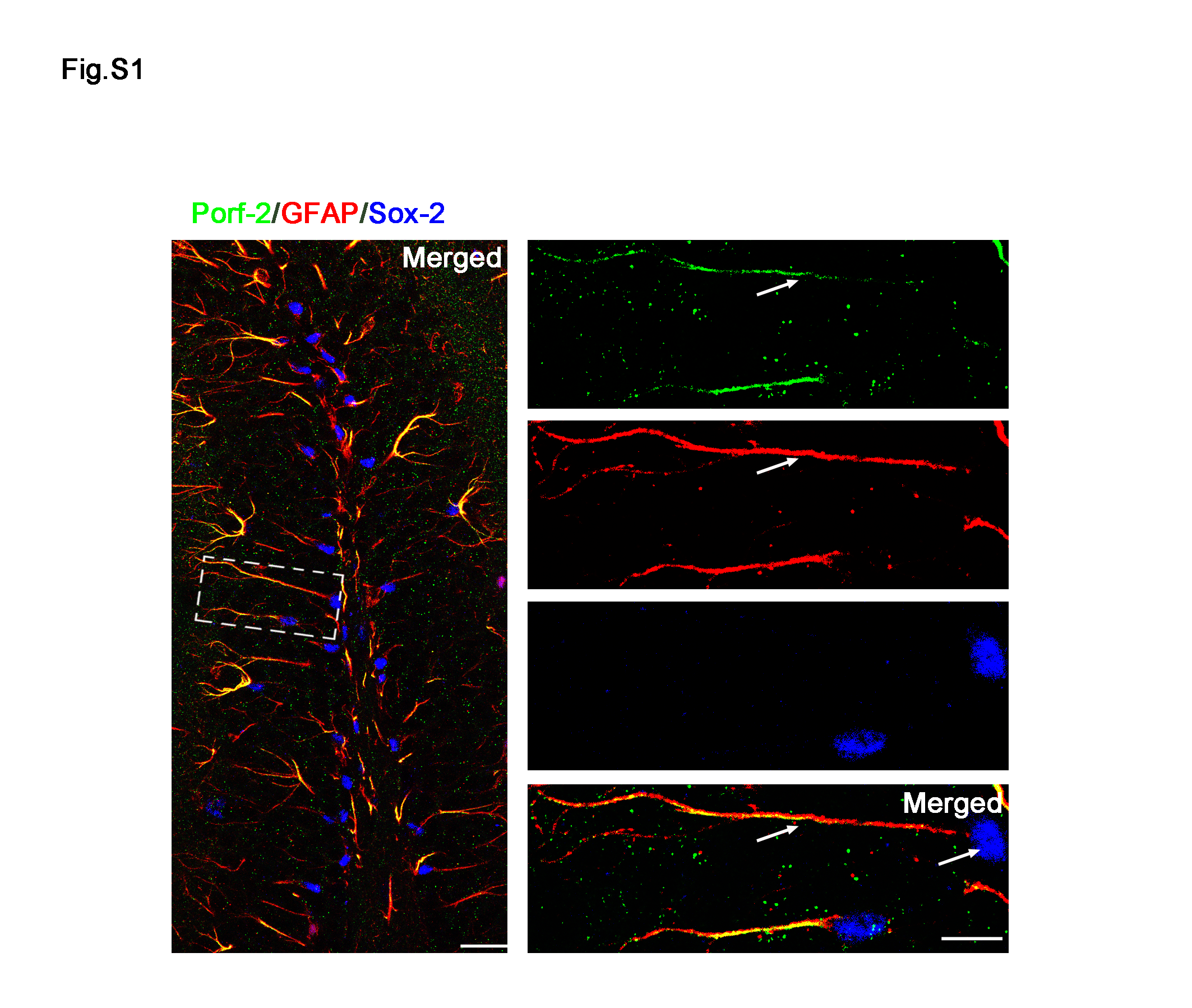

Supplement: Figure S1 — Porf-2 is expressed in NSCs of subgranular zone in DG. Expression of of porf-2, Sox-2, GFAP were visualized by immunolabeling with indicated antibody in DG area of brain slice. The boxes in left panel are shown at higher magnification in right panels. The white arrowhead indicated the colocalization of porf-2 with GFAP+/Sox-2+ cell. Scale bars: 25 μm in the left panel and 10 μm in the right panel. [file Image1.TIF]

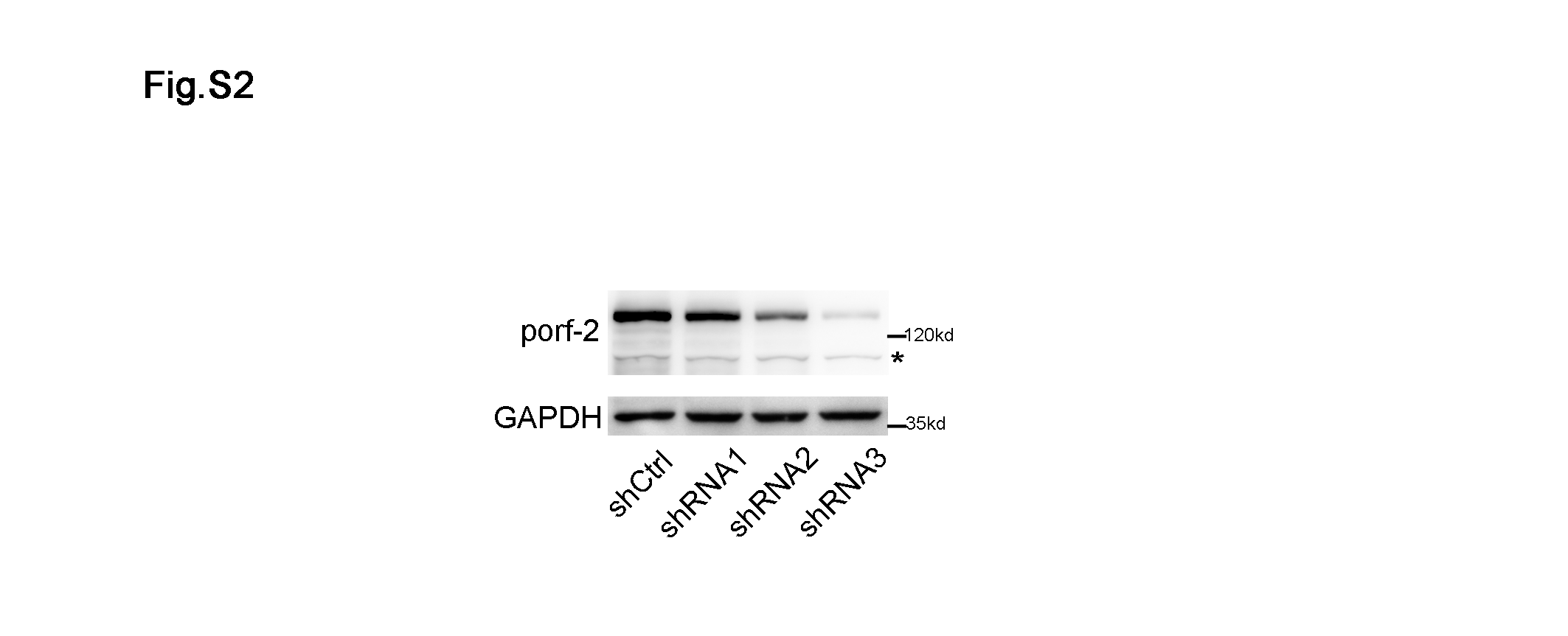

Supplement: Figure S2 — Porf-2 was knocked down by shRNA. Porf-2 knock-down was confirmed by Western Blot analysis. The symbol* indicated the non-specific band smaller than 120 KD. GAPDH was used as a loading control. [file Image2.TIF]

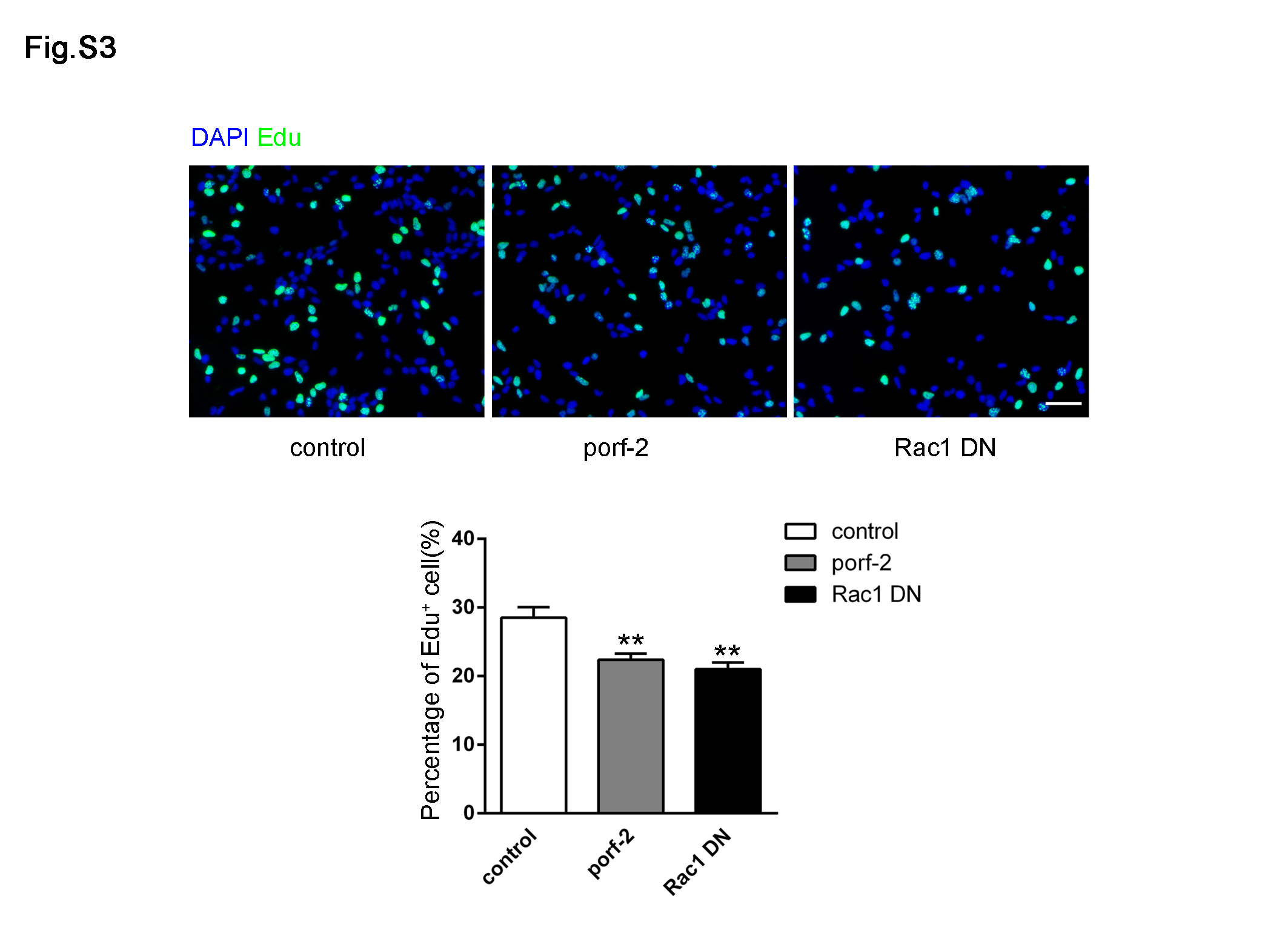

Supplement: Figure S3 — Rac1 DN showed the same phenotype as Porf-2 expression on NSC proliferation. Representative image of Edu positive cells in each group. The total NSC number was counted by DAPI. Quantification of the percentage of Edu positive cell number in each group. Scale bar: 50 μm. Data are mean ± SEM (n = 4). **P < 0.01 vs. control. [file Image3.TIF]
